# Stochastic learning and extremal-field map based autonomous guidance of low-thrust spacecraft

**DOI:** 10.1038/s41598-022-22730-y

**Published:** 2022-10-22

**Authors:** Sandeep K. Singh, John L. Junkins

**Affiliations:** 1 Mechanical, Aerospace and Nuclear Engineering, Rensselaer Polytechnic Institute, 110 8th Street, Troy, NY 12180 USA; 2 Department of Aerospace Engineering, Texas A & M University, College Station, TX 77843 USA

**Keywords:** Engineering, Aerospace engineering

## Abstract

A supervised stochastic learning method called the Gaussian Process Regression (GPR) is used to design an autonomous guidance law for low-thrust spacecraft. The problems considered are both of the time- and fuel-optimal regimes and a methodology based on “perturbed back-propagation” approach is presented to generate optimal control along neighboring optimal trajectories which form the extremal bundle constituting the training data-set. The use of this methodology coupled with a GPR approximation of the spacecraft control via prediction of the costate *n*-tuple or the primer vector respectively for time- and fuel-optimal trajectories at discrete time-steps is demonstrated to be effective in designing an autonomous guidance law using the open-loop bundle of trajectories to-go. The methodology is applied to the Earth-3671 Dionysus time-optimal interplanetary transfer of a low-thrust spacecraft with off-nominal thruster performance and the resulting guidance law is evaluated under different design parameters using case-studies. The results highlight the utility and applicability of the proposed framework with scope for further improvements.

## Introduction

Over recent decades, autonomy has emerged as an intense field of research for a wide range of engineering applications like robotics and autonomous vehicles. With the advancements in computational capability both online and offline, available to researchers today, significant advancements have been made towards design and development of intelligent systems. More recently, autonomous systems concepts have been applied in the field of advanced space mission design. Autonomous guidance, in general, can be used in this context for applications such as, low-thrust orbital transfer, station-keeping, re-planning etc. The motivation behind designing a space-system with an inbuilt autonomy can be best understood by studying the details of the Dawn Mission^1^. The aim of this highly successful mission was to study two distant proto-planets namely, Vesta and Ceres and the total time of flight for the spacecraft was 8 years (2007–2015). Remarkably, the flown spacecraft trajectory required $\approx$ 5.865 years of ion engine thrusting and collected more than 172 Gigabytes of science data. An important facet that contributed to the mission’s success was the re-planning of the spacecraft trajectory “to-go” was required due to a combination of navigation errors and engine off-nominal performance, literally 100s of “to-go” trajectory re-design iterations were required during the mission. These re-design iterations were done using human-analyst aided legacy trajectory optimization tools. The substantial ground support required to do 100’s of trajectory -to-go optimization for the DAWN mission underscores the need of an in-built autonomy aboard future interplanetary spacecraft in order to fly to the desired target orbit or rendezvous with the desired space object in the presence of unavoidable off-nominal behavior due to erroneous state-estimation or engine performance. The utilization of autonomous navigation advances in on-board sensing and especially, more robust trajectory optimization and guidance algorithms are fundamental enablers for future autonomous deep-space guidance.

Interplanetary mission design and guidance traditionally begins with the generation of a nominal trajectory on the ground i.e., pre-flight. During flight, the on-board guidance and control system takes the current best state estimates of the spacecraft computed through orbit determination algorithms as input and tracks the nominal trajectory through the requisite control. Typically, existing on-board guidance laws are designed based on a polynomial representation of the thrust with the coefficients optimized to cancel any deviations from the nominal trajectory^2^. Many advancements towards spacecraft autonomous guidance, navigation and control applications to tackle deviation in target conditions have also been made. For instance, Jet Propulsion Laboratory (JPL) uses ‘Autonomous Navigation (AutoNav)’ for all camera-equipped comet encounter missions flown by NASA like Borrelly, Wild 2, Tempel 1 and Hartley 2^3^. AutoNav, based on image processing, orbit determination and control computation, has been hugely successful in tracking comet and asteroid nuclei. However, the underlying issue with guidance laws derived on the ground is that they do not work for unexpectedly large deviations which can only be characterized in real-time. On the other end of this spectrum, a completely real-time computation of the reference trajectory is beyond the scope of the conventional on-board computers due to the convergence challenges in solving the two point boundary value problems (TPBVPs).

Machine learning (ML) algorithms based on neural networks (NNs) and deep neural networks (DNNs) have gained a lot of traction recently as promising research avenues for autonomous/ real-time spacecraft applications. Sanchez-Sanchez et al.^4^ studied the use of DNNs in the context of planetary landings. Similarly, Cheng et al. studied real-time optimal control for irregular asteroid landings^5^ and for spacecraft orbit transfer using Multi-scale DNNs^6^. In another interesting ML application, Li et al. used NNs in the context of designing autonomous, time-optimal, low-thrust interplanetary transfers^7,8^. In these works, NNs were trained to approximate co-states, optimal thrust and the value function of the optimal interplanetary transfers. The problem of generating/obtaining a well-populated and well-behaved data-set was solved for fuel-optimal problems recently by Izzo et al.^9^ where they used a “backward propagation of optimal examples approach” to generate neighboring optimal trajectories for the Earth-Venus mass-optimal interplanetary transfer of low-thrust spacecraft to generate data which fed into a DNN framework and provided real-time guidance laws. While the methodology of generating neighboring optimal trajectories was effective for mass-optimal trajectories in this work, a similar approach to generate neighboring time-optimal trajectories was not presented. Additionally, the choice of learning method was strictly limited to DNNs only which require a significantly large data-set to give reasonably accurate results. The present paper was in-part motivated by Izzo et al.^9^.

Application of ML approaches to real-time spacecraft guidance has emerged as an important research area recently. Furfaro et al.^10^ studied the use of Deep Neural Networks (DNNs) in the context of autonomous luanr landing. The missed thrust problem has also been tackled using a NN framework using reinforcement learning (RL) by Rubinsztejn et al.^11,12^. Federici et al.^13^ studied Deep Learning techniques for autonomous spacecraft guidance during proximity operations. Zavioli and Federici also studied RL for interplanetary trajectory design in^14^. More recently, an advanced machine learning framework called “meta-reinforcement learning” has been studied by Federici et al.^15^, Scorsoglio et al.^16^. The method has been demonstrated to have an improved performance as compared to the “more traditional” deep-learning approaches. Optimal control problems in the context of spacecraft trajectory design has also been studied recently using physics-informed neural networks (PINNs) by D’Ambrosio et al.^17^.

In order to generate a guidance law using any supervised learning method, the bundle of neighboring optimal trajectories must include the expected worst deviations from the pre-designed nominal trajectory of the spacecraft. If this is not the case, the predicted guidance law will likely be erroneous due to the nature of supervised learning because extrapolations outside the support of training information is always dangerous. Additionally, for on-board computation, data storage is another challenge and learning methods which work well with smaller data-sets need to be explored and methods that associate some uncertainty metric with the predictions are preferred. Gaussian process regression (GPR) is one such tool, which can be used for supervised learning to predict target value(s) given some observations. The approach is recursive and adaptive with the implicit locations and the shape of the interpolation functions being adapted through training data. More importantly, the method also produces an uncertainty estimate for the predictions which enables intelligent and adaptive input sampling. In essence, closing the loop between training data generation and the uncertainty of current prediction is a critical feature of the GPR based guidance approach we present. GPR was first proposed by Danie G. Krige^18^ to estimate gold distribution. Since then variations of these ideas have been used in several other fields like airfoil design^19^ and aerodynamic coefficient of a spaceplane^20^. Some other works demonstrate the effectiveness of GPR based learning methods as an I/O training model for regression in a wide variety of applications ranging transmission spectroscopy^21^, evolution of micro-structure statistics in super alloys^22^, probabilistic modelling of wind-turbine power curves^23^ and bearing degradation assessment^24^. In the field of astrodynamics, Shang and Liu^25^ assessed accessibility of Main-Belt asteroids by predicting the optimal bi-impulsive $\Delta$V costs to rendezvous with the asteroids via a GPR model trained on the family of such transfers to rendezvous states in a domain spanning the known main-belt asteroid region. Singh et al.^26^ later demonstrated the effectiveness of a multi-input multi-output GPR in predicting the optimal family of Lambert transfer trajectories for cis-lunar transfers from Earth to a periodic $L_1$ Halo orbit; leveraging invariant manifolds.

The GPR approach considers inference directly in function space. It is considered to be a non-parametric regression method and is computationally efficient while handling I/O relations in regression problems. Due to being based on Bayesian inference, it implicitly provides an estimate of the mean as well as the covariance of the predicted outputs. Other learning-based approximation methods can only provide an explicitly computed statistical covariance measure of the prediction errors during post-processing. In this paper, we build on the work of Izzo et al. by providing a methodology to generate the bundle of extremal trajectories for both time- and fuel-optimal low thrust transfer trajectories using the “backward propagation of optimal examples” approach for Earth-Dionysus transfer. By varying terminal co-states, a bundle of optimal trajectories neighboring the nominal trajectory can be readily generated. The size of the terminal co-state perturbations can be tuned such that the dispersion of the neighboring optimal trajectories are comparable to the worst-case navigation errors and off-nominal thrust errors. The sensitivity of perturbations in terminal co-states on the nature of the trajectory bundle is discussed and illustrated in the developments that follow. Using the generated bundle of optimal trajectories, time-sliced hyper-surfaces in the augmented state-costate space are used to define the domain for training individual multi-input multi-output (MIMO) GPR models. The models are used to predict co-states at each time-step as a function of the state-variables which can be directly used to determine the optimal control for any state along the respective ‘to-go’ trajectories. The applicability and advantage of using the trained model in terms of re-planning trajectories is demonstrated using the example of an off-nominally performing thruster of a spacecraft engine flying a nominal time-optimal trajectory from Earth to Dionysus. A multitude of case studies have been performed to explore the effect of frequency and location of way-points (trajectory re-planning instances) on the performance of the resulting autonomous guidance algorithm.

In summary, the key contributions of the paper are (1) describing a robust methodology to generate an extremal field map of optimal trajectories which neighbor a nominal trajectory for both time- and fuel-optimal trajectories, (2) proposing and documenting a robust algorithm to train multi-input multi-output GPR model based on the generated extremal bundle as well as describing an appropriate choice of the kernel function and (3) demonstrating the feasibility of employing Gaussian process regression as a machine learning tool for multiple re-planning of deep space missions. The remainder of this paper is structured as follows: “Preliminaries” section covers the preliminaries and introduces GPR as well as describes the various kernels. Section “Trajectory optimization problem” describes the trajectory optimization problem and provides analytical derivations of the optimal control for time- and fuel-optimal trajectories using the indirect formulation of the problem. Section “Nominal trajectory” gives details of the nominal trajectory for the Earth-Dionysus interplanetary transfer problem. Section “Generation of extremal trajectory bundles” lists the procedure for generating extremal trajectory bundles for time- and fuel-optimal trajectories and describes the respective algorithms. Section “Results: trajectory re-planning—off-nominal thrust” includes results where the GPR models were trained for a specific off-nominal thruster problem and provides results from case studies. Section “Conclusion” is the conclusion.

## Preliminaries

### Gaussian process regression

A Gaussian process is defined as a collection of random variables, any finite number of which have a joint Gaussian distribution^27^. It is completely specified by its mean function and the covariance function. For instance, consider a real process $f({{\textbf {x}}})$ with the mean function defined as $m({{\textbf {x}}}) = {\mathbb {E}}[f({{\textbf {x}}})]$ and the covariance function as $k({{\textbf {x}}},{{\textbf {x}}}') = {\mathbb {E}}[(f({{\textbf {x}}}) - m({{\textbf {x}}}) (f({{\textbf {x}}}') - m({{\textbf {x}}}'))]$. The Gaussian process is then written as $f({{\textbf {x}}}) \sim {{\mathcal {G}}}{{\mathcal {P}}}(m({{\textbf {x}}}), k({{\textbf {x}}},{{\textbf {x}}}'))$.

Gaussian process regression (GPR) is a supervised learning method, which makes use of the marginalization property of Gaussian processes. It is primarily a non-parametric, Bayesian approach, which infers a probability distribution over all possible functions, instead of determining exact parameters of some set of basis function as is the case with traditional approximation techniques. Random functions are typically drawn from a prior distribution, which is a zero mean Gaussian distribution with the covariance function evaluated at input points. The zero mean assumption does not lead to any loss of generality. There are many valid choices for covariance function, with the squared exponential (SE) and the rational quadratic (RQ) functions being the most popular and able to cover a vast majority of applications.

GPR uses Bayes’ rule to incorporate training data to compute a posterior distribution using Likelihood and the assumed prior distribution. This procedure is known as conditioning the joint Gaussian prior distribution on observations. Therefore, the joint distribution of the training outputs, ${{\textbf {f}}}$, and the testing outputs ${{\textbf {f}}}_{*}$ according to the prior is expressed as

1 $$\begin{aligned} \begin{bmatrix} {{\textbf {f}}} \\ {{\textbf {f}}}_{*} \end{bmatrix} \sim {\mathcal {N}} \ \Bigg ( {{\textbf {0}}}, \begin{bmatrix} K(X,X) &{} \quad K(X,X_{*}) \\ K(X_{*},X) &{} \quad K(X_{*},X_{*}) \end{bmatrix} \Bigg ), \end{aligned}$$

where ${\mathcal {N}}(.)$ denotes the normal distribution notation and if there are *n* training points and $n_*$ testing points then $K(X,X_{*})$ denotes the $n \times n_*$ matrix of covariances evaluated at all training and test pair of points. The other entries, i.e., *K*(*X*, *X*), $K(X_{*},X)$ and $K(X_{*},X_{*})$ are evaluated similarly. Note that while making predictions, we consider the joint distribution of both the training and prediction data-sets.

In order to generate the posterior distribution over functions, the joint prior distribution above must be restricted to contain only those functions which agree with the observed data-points, i.e., incorporate the information from the training set. The distribution of the ${{\textbf {f}}}_*$ conditioned on ${{\textbf {f}}}$, *X* and $X_*$ is expressed as

2 $$\begin{aligned} {{\textbf {f}}}_*|{{\textbf {f}}},X,X_* \sim {\mathcal {N}} \ (K(X_*,X)K(X,X)^{-1}{{\textbf {f}}}, K(X_*,X_*) - K(X_*,X)K(X,X)^{-1}K(X,X_*)). \end{aligned}$$

Thus, ${{\textbf {f}}}_*$ can be sampled from this joint posterior distribution. This gives the statistically inferred predicted outputs at some desired inputs when the prediction inputs are used instead of the testing set.

#### Optimization of the hyperparameters

As mentioned before, a multitude of possible covariance functions exist. These families of functions are typically characterized by a number of free hyperparameters, which need to be determined. The determination of an appropriate covariance function followed by computation of the associated hyperparameters falls under the *training* of a Gaussian process.

For a noise-free model, the squared exponential function for instance can be parameterized in terms of hyperparameters as

3 $$\begin{aligned} k({{\textbf {x}}}_p,{{\textbf {x}}}_q) = \sigma _f^2 \ \text {exp} \ \left( -\frac{1}{2}({{\textbf {x}}}_p - {{\textbf {x}}}_q)^{T} Q ({{\textbf {x}}}_p - {{\textbf {x}}}_q)\right) , \end{aligned}$$

where $\varvec{\Phi } = (\{Q\},\sigma _f^2)$ is the vector of hyperparameters, $\sigma _\text {f}^2$ regulates the amplitude of the output distribution and ${{\textbf {x}}}_\text {p}$, ${{\textbf {x}}}_\text {q}$ are samples of input vectors individually spanning the whole input space. The most common choice for the matrix *Q* is $\text {diag}({{\textbf {l}}})^{-2} I$ where ${{\textbf {l}}}$ is a vector of positive values and are typically analogous with characteristic length scales. In general, a numerical optimization methodology is incorporated to determine these set of hyperparameters with the objective that the likelihood of the training outputs given the regression model is maximized. The log likelihood is defined as

4 $$\begin{aligned} {\mathbb {L}}(\varvec{\Phi }) = \text {log} \ p({{\textbf {f}}}|X,\varvec{\Phi }) = -\frac{1}{2} \ {{\textbf {f}}}^{T}K^{-1}{{\textbf {f}}} - \frac{1}{2} \ \text {log} \ |K| - \frac{n}{2} \ \text {log} \ 2\pi . \end{aligned}$$

Note that the log-likelihood is a function of $\varvec{\Phi }$ and ($X,{{\textbf {f}}}$). Thus, given the training data and a choice of covariance function, the optimal set of hyperparameters ($\varvec{\Phi }^*$) can be computed as

5 $$\begin{aligned} \varvec{\Phi }^{*} = \text {arg max}_{\varvec{\Phi }} {\mathbb {L}}(\varvec{\Phi })|_{X,{{\textbf {f}}}}. \end{aligned}$$

For instance, MATLAB routine *fminsearch* can be used with an initial guess for the set of hyperparameters to minimize the negative log likelihood in order to optimize the hyperparameters. It is hereby noted that, the initial guess is key due to the existence of various local extrema for a multivariable optimization problem. It is therefore recommended to use a ‘population-based algorithm’ like particle-swarm optimization (PSO) or evolutionary algorithms in conjunction with *fminsearch* to find the global optimum in a specified domain. The results presented in the paper are obtained by utilising such a hybrid global-local algorithm, ‘PSO-*fminsearch*’ optimization algorithm.

The choice of covariance function for the problems considered in this paper is a modified RQ function. The classical RQ function is stationary, i.e., depends solely on distances between samples in $D-dim$ Euclidean space. In order to cover the non-stationarity due to the different orbital elements, which serve as inputs to the problems discussed later, an automatic relevance determination (ARD) distance measure is integrated into the covariance function and is now expressed as,

6 $$\begin{aligned} k({{\textbf {x}}}_p,{{\textbf {x}}}_q) = \sigma _f^2 \ \text {exp} \Bigg ( 1 + \frac{({{\textbf {x}}}_p - {{\textbf {x}}}_q)^T {\mathcal {Q}} ({{\textbf {x}}}_p - {{\textbf {x}}}_q)}{2\alpha }\Bigg )^{-\alpha }, \end{aligned}$$

where $\sigma _f^2$ is analogous to an amplitude measure of the output distribution, $\alpha$ represents the shape of the signal and ${\mathcal {Q}}$ is a symmetric matrix of the characteristic length scale in the different input dimensions and determines the relevance of a particular input to the covariance function and is expressed for a $D-\textit{dim}$ input system as,

7 $$\begin{aligned} {\mathcal {Q}} = \text {diag}\Bigg (\frac{1}{l_1^2}, \frac{1}{l_2^2}, \frac{1}{l_3^2},....., \frac{1}{l_D^2}\Bigg ) \ \ \ \ \text {and} \ \ \ \ \varvec{\Phi } = [l_1^2,l_2^2,l_3^2,...., l_D^2, \sigma _f^2, \alpha ]. \end{aligned}$$

The characteristic lengths, appear as an inverse square in the expression and are responsible for signifying the heuristic fact—*outputs for system inputs that are closer to each other will be more highly correlated than those where inputs are further apart.*

#### Multiple-input multiple-output (MIMO) GPR

In the context of this paper, the trained model is supposed to predict either the primer vector, $\varvec{\lambda }_p$ in the case of a time-optimal transfer, or the full costate *n*-tuple, $\varvec{\Lambda }$, in the case of a fuel-optimal transfer. The state-space for a typical trajectory design problem formulated using the modified equinoctial elements (MEEs) is: ${\mathbf {X}} = [p,f,g,h,k,L,m]^T$ where *p*, *f*, *g*, *h*, *k*, *L* are the MEEs and *m* denotes the mass. The costate n-tuple, $\varvec{\Lambda } = [\lambda _p,\lambda _f,\lambda _g,\lambda _h,\lambda _k,\lambda _L,\lambda _m]^T$ include the adjoint parameters associated with each state. Therefore, ${\mathbf {X}} \in {\mathbb {R}}^7$, $\varvec{\lambda }_p \in {\mathbb {R}}^3$ and $\varvec{\Lambda } \in {\mathbb {R}}^7$.

For this paper, the best current estimate of the spacecraft state is assumed to be available from the orbit determination algorithm and includes the navigation errors as well as errors due to other unmodeled/unanticipated effects in deviation from the nominal. The purpose of the learning model is to accept this data as input and provide a prediction of the costate n-tuple or the primer vector based on the data from the extremal optimal bundle that it has been trained on.

The respective mapping functions of the regression model, which is able to predict all the components of $\varvec{\Lambda }$ or $\varvec{\lambda }_p$, namely, ($f_1 \& f_2$) can be expressed as:

8 $$\begin{aligned} \begin{aligned} f_1: {\mathbb {R}}^7&\longrightarrow {\mathbb {R}}^7 \ \ i.e., \ \ {\mathbf {X}} \longrightarrow \varvec{\Lambda },\\ f_2: {\mathbb {R}}^7&\longrightarrow {\mathbb {R}}^3 \ \ i.e., \ \ {\mathbf {X}} \longrightarrow \varvec{\lambda }_p, \end{aligned} \end{aligned}$$

This GPR model is termed as a MIMO GPR and referred to as such henceforth. In addition to providing higher-fidelity predictions, another advantage of using a MIMO GPR instead of many multi-input single output (MISO) GPRs is the use of the information embedded in the correlations among the outputs for training purposes. As discussed before, the covariance functions are solely dependent on the inputs. On the other hand, in case the desired prediction include multiple outputs, the knowledge of correlation between the outputs is lost while using multiple MISO GPR models. The same scenario occurs, albeit implicitly, when the desired ‘single’ output is a composite function of the multiple individual outputs, as is the case discussed above. Therefore, using a MIMO GPR instead of multiple MISO GPRs is expected to provide more accurate predictions as shown in^28,29^.

## Trajectory optimization problem

Consider the motion of a spacecraft from Earth to a desired heliocentric orbit under the effect of the Sun’s gravitational attraction in the International Celestial Reference Frame (ICRF). The spacecraft’s mass is represented by *m* and is equipped with an engine of specific impulse ($\text {I}_\text {sp}$), maximum thrust ($\text {T}_\text {max}$). The choice of element-set to represent the equations of motion and subsequent formulation of the optimization problem is the MEEs. The spacecraft state vector is represented by ${\mathbf {X}}$ while the costate vector is represented by $\varvec{\Lambda }$. The subsequent sections describe the indirect formulation of the optimal control problem as well as the associated boundary conditions for the resulting TPBVP for both time- and fuel-optimal problems.

### Equations of motion

The motion of the spacecraft under the effect of the Sun’s gravitational pull in the heliocentric ICRF can be described by the following set of equations for $\{{\mathbb {X}}\} = \{{\mathbf {X}}\} \ {\setminus } \ m$,

9 $$\begin{aligned} \dot{{\mathbb {X}}}&= \varvec{b} + \frac{T_\text {max}}{m_0 \ m \ g_0} {\mathbb {M}} \varvec{\delta _T} u,&{\dot{m}} =&-\frac{T_{\text {max}}}{m_0 \ c} u, \end{aligned}$$

where $\varvec{\delta _T}$ is the unit vector in the thrust direction, u is the engine throttle input and $\text {u} \in [0,1]$, and $c = \text {I}_\text {sp}/(\sqrt{\frac{AU}{g_0^3}})$ denotes the effective exhaust velocity. Note that in order to scale the problem, *P* is normalized by AU (1AU = $1.496 \times 10^{-8} \ \text {km}$), mass is normalized by $m_0$, while *t* is normalized by $\sqrt{AU/g_0}$, $m_0$ is the initial mass and $g_0$ is the reference value of earth’s surface gravity used in traditional specific impulse ($\text {I}_\text {sp}$) definition and is equal to 9.80665 m/s$^2$. In the equations above, $T_\text {max}$ is the (constant) maximum thrust provided by the spacecraft engine. The vector $\varvec{b}$ and matrix ${\mathbb {M}}$ are defined as

10 $$\begin{aligned} {\mathbb {M}} =&\sqrt{\frac{P}{\mu }} \begin{bmatrix} 0 &{} \quad \frac{2P}{W} &{} \quad 0\\ \sin {L} &{} \quad \cos {L} + \frac{e_x + \cos {L}}{W} &{} \quad -\frac{Z e_y}{W}\\ -\cos {L} &{}\quad \sin {L} + \frac{e_y + \sin {L}}{W} &{}\quad \frac{Z e_x}{W} \\ 0 &{}\quad 0 &{} \quad \frac{C \cos {L}}{2W} \\ 0 &{} \quad 0 &{} \quad \frac{C \sin {L}}{2W} \\ 0 &{}\quad 0 &{} \quad \frac{Z}{W} \end{bmatrix},&\quad \varvec{b} =&\begin{bmatrix} 0\\ 0\\ 0\\ 0\\ 0\\ \sqrt{\mu P}\Big (\frac{W}{P}\Big )^2 \end{bmatrix}, \end{aligned}$$

where the scalars $W = 1 + e_x \cos {L} + e_y \sin {L}$, $Z= h_x \sin {L} - h_y$ and $C= 1 + h_x^2 + h_y^2$.

### Time-optimal trajectories

The first class of problems considered in the paper belong to the time-optimal, free final time ($t_f$) and free final true longitude type transfers ($L_f = L(t_f)$). Introducing $\{\varvec{\Gamma }\} = \{\varvec{\Lambda }\} \ {\setminus } \ \lambda _m$ and the performance index $J_T = t_f$, the scalar Hamiltonian for this class of problems is defined as the matrix product,

11 $$\begin{aligned} H_\text {T} = \varvec{\Lambda }^T \dot{{\mathbf {X}}}. \end{aligned}$$

The costate dynamics are obtained using the Euler–Lagrange equation, $\dot{\varvec{\Lambda }} = -[\partial H_\text {T}/\partial {\mathbf {X}}]^T$. The optimal thrust direction should be opposite to the direction of ${\mathbb {M}}^T \varvec{\Gamma }$ to minimize the Hamiltonian in accordance with the minimum principle i.e.,

12 $$\begin{aligned} \varvec{\delta _T}^{*} = -\frac{{\mathbb {M}}^T \varvec{\Gamma }}{\Vert {{\mathbb {M}}^T \varvec{\Gamma }} \Vert }. \end{aligned}$$

The eight unknowns for the minimum-time orbit-transfer TPBVP are $\varvec{\Lambda }(t_0)$ and $t_{f}$. Eight final conditions must be satisfied including five state final conditions and the three transversality conditions associated with ‘free final time’ expressed as $1 + H_\text {T}({t_{f}}) = 0$, ‘free final mass’ expressed as $\lambda _m(t_f) = 0$ and ‘free final true longitude’ expressed as $\lambda _L(t_f) = 0$. The solution to the aforementioned TPBVP gives a minimum-time extremal transfer trajectory. Note that $u^*(t) = 1$ for minimum-time problems i.e., the thrust is continuous and always ‘ON’.

### Fuel-optimal trajectories

The paper also considers the fuel-optimal, free final time ($t_f$) and free final true longitude type transfers ($L_f = L(t_f)$). The performance index $J_f$ is defined in terms of the propellant mass $m_\text {p}$ and the throttling input, $\sigma$, as

13 $$\begin{aligned} J_{\text {MF}} = m_\text {P} = m(t_0) - m(t_f) = -\int _{t_0}^{t_f} {\dot{m}} \ dt = \frac{T_\text {max}}{m_0 \ c} \int _{t_0}^{t_f} \sigma \ dt. \end{aligned}$$

The scalar Hamiltonian for this class of problems ($H_\text {f}$) is defined as,

14 $$\begin{aligned} H_{\text {F}} = \frac{T_\text {max}}{m_0 \ c} \sigma + \varvec{\Lambda }^T \dot{{\mathbf {X}}}. \end{aligned}$$

Substituting the value of $\dot{{\mathbf {X}}}$ in Eq. (14), with $T_\text {max}$ in the minimum-time formulation replaced by $T_\text {max} \ \sigma$ for the minimum-fuel formulation to account for engine throttling, the following is obtained for the Hamiltonian

15 $$\begin{aligned} H_\text {F} = \frac{T_\text {max}}{m_0 \ c} \sigma + \varvec{\Gamma }^T \varvec{b} + \frac{T_\text {max}}{m_0 \ m \ g_0} \ \sigma \varvec{\Gamma }^T {\mathbb {M}} \varvec{\delta _T} - \lambda _m \frac{T_\text {max}}{m_0 \ c} \sigma . \end{aligned}$$

Since $H_\text {F}$ is a bi-linear function of both controls, $\varvec{\delta _T}$ and $\sigma$, the strong form of the optimality conditions, (i.e., $\partial H_\text {F}/\partial \varvec{\delta _T} = 0$ and $\partial H_\text {F}/\partial \sigma = 0$) are not applicable. Invoking Pontryagin’s Minimum Principle, optimal control inputs can be obtained upon inspecting the $3^{rd}$ term of equation (15), since $\sigma$ is inherently non-negative as,

16 $$\begin{aligned} \varvec{\delta _T}^{*} =&-\frac{{\mathbb {M}}^T \varvec{\Gamma }}{\Vert {{\mathbb {M}}^T \varvec{\Gamma }} \Vert },&\sigma ^{*} \in&\ \text {arg} \min _{0 \le \sigma \le 1} H_\text {F}. \end{aligned}$$

In order to obtain $\sigma ^{*}$, upon substituting $\varvec{\delta _T}^*$ into the Hamiltonian, the terms that are affine in the throttle, $\sigma$, need to be minimized. This function, named $\sigma _\text {aff}$, is mathematically expressed as,

17 $$\begin{aligned} {\sigma _\text {aff}} = -\frac{T_\text {max}}{m_0 \ c} \sigma \Bigg (\frac{c \varvec{\Gamma }^T {\mathbb {M}} \varvec{\delta _T}^{*}}{m} + \lambda _m - 1 \Bigg ). \end{aligned}$$

Define the term in the bracket in Eq. (17) as a thrust switching function (SF) as

18 $$\begin{aligned} \text {SF} = \frac{c \varvec{\Gamma }^T {\mathbb {M}} \varvec{\delta _T}^{*}}{m} + \lambda _m - 1, \end{aligned}$$

and assuming an absence of intermittent singular arcs (SF = 0 for finite time intervals), the optimal value of the throttling parameter, $\sigma ^*$, can be defined as a function of SF,

19 $$\begin{aligned} \sigma ^* (\text {SF})= {\left\{ \begin{array}{ll} 1, &{} \quad \text {if}\ \text {SF} \ > \ 0 \\ 0, &{} \quad \text {if}\ \text {SF} \ < \ 0. \end{array}\right. } \end{aligned}$$

Solving optimal control problems with bang-off-bang type of engine throttling (which is a characteristic of minimum-fuel type transfers) is difficult primarily because of the discontinuous nature of the control. In order to make the resulting TPBVPs amenable to numerical treatment, a smoothing parameter, $\rho$, is introduced using the hyperbolic tangent smoothing (HTS) method^30^. The generalized HTS for any admissible control input is defined as^31^

20 $$\begin{aligned} \sigma ^* (\text {SF},\rho ) = \frac{1}{2} \Bigg [ (\sigma _\text {U} + \sigma _\text {L}) + (\sigma _\text {U} - \sigma _\text {L})\tanh \Bigg ({\frac{\text {SF} - \text {SF}_s}{\rho }} \Bigg ) \Bigg ], \end{aligned}$$

where $\sigma _\text {U}$ and $\sigma _\text {L}$ are the upper and lower bounds for the throttling parameter respectively and $(\sigma _\text {L},\sigma _\text {U}) \equiv (0,1)$. $\text {SF}_s$ is the switching point and for the class of transfers studied in this paper, $\text {SF}_s = 0$. Therefore, the smoothed throttling input is

21 $$\begin{aligned} \sigma ^* (\text {SF},\rho ) = \frac{1}{2} \Bigg ( 1 + \tanh \Bigg ({\frac{\text {SF}}{\rho }}\Bigg ) \Bigg ). \end{aligned}$$

The costate dynamics are obtained using the first order necessary conditions in the form of the Euler–Lagrange equation, $\dot{\varvec{\Lambda }} = -[\partial H_\text {F}/\partial {\mathbf {X}}]^T$. The eight unknowns for the minimum-fuel orbit-transfer TPBVP are $\varvec{\Lambda }(t_0)$ and $t_{f}$. Eight final conditions must be satisfied including five state final conditions and the three transversality conditions associated with ‘free final time’ expressed as $1 + H_\text {F}({t_{f}}) = 0$, ‘free final mass’ expressed as $\lambda _m(t_f) = 0$ and ‘free final true longitude’ expressed as $\lambda _L(t_f) = 0$. A parametric continuation is performed on $\rho$ till the control profile tends to the desired bang-off bang profile. The solution to the aforementioned TPBVP gives a minimum-fuel extremal transfer trajectory. It is hereby reiterated that the thrust is discontinuous and not always ‘ON’, unlike the time-optimal case.

## Nominal trajectory

**Figure 1:**
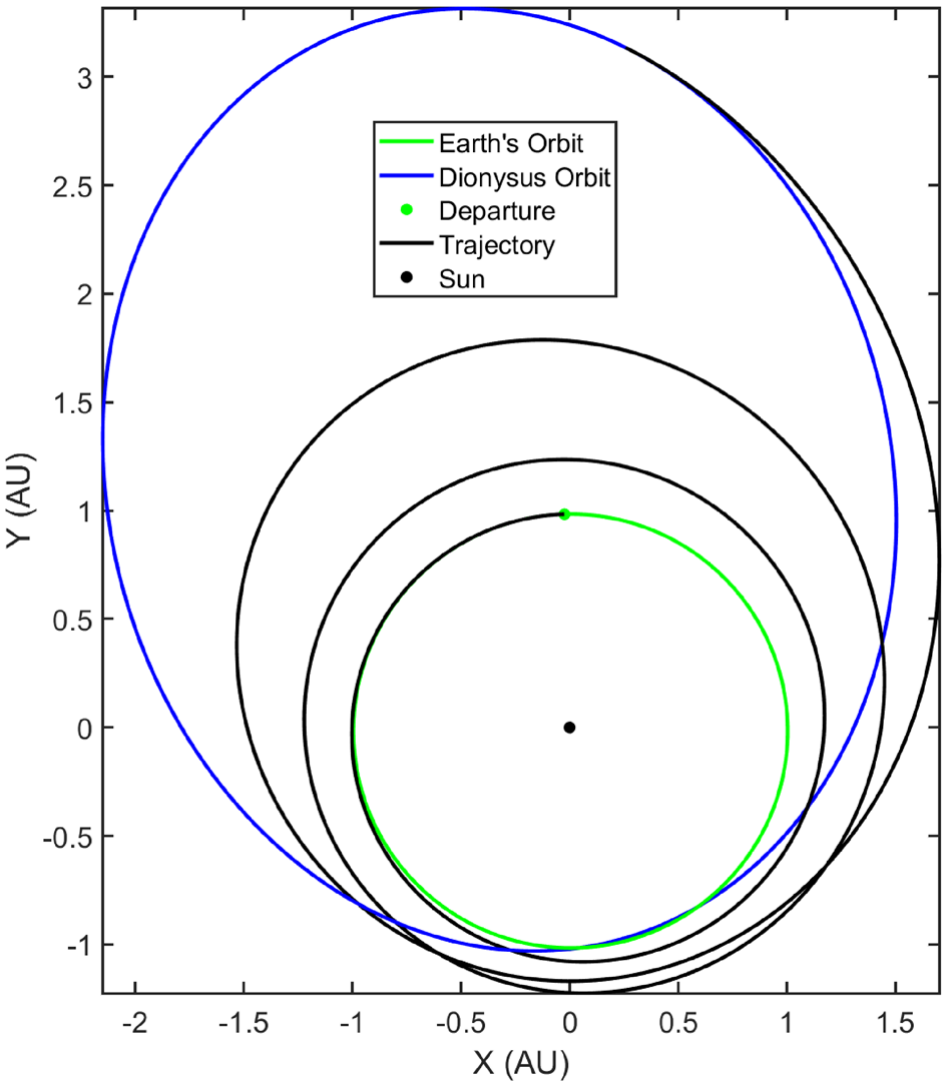
Earth-3671 dionysus time-optimal nominal transfer.

**Figure 2:**
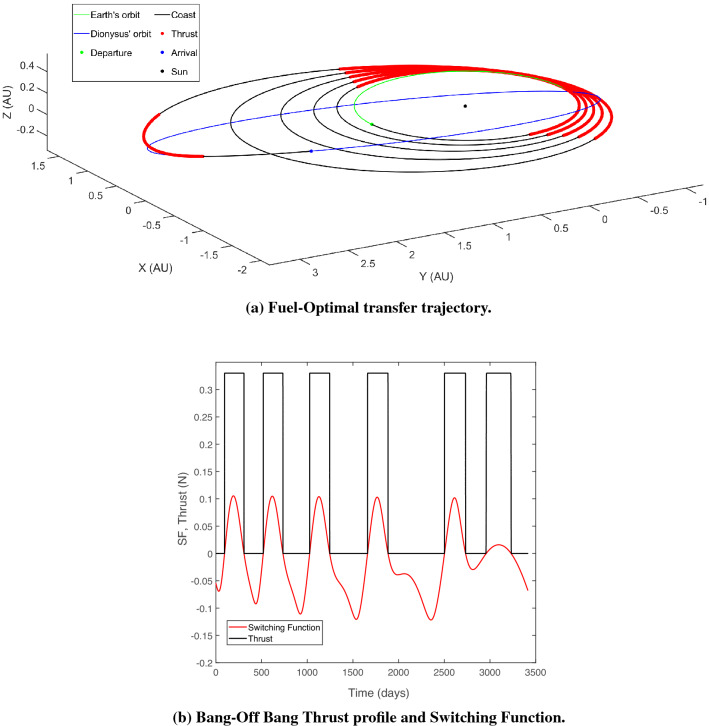
Earth-3671 dionysus fuel-optimal nominal transfer.

For this paper, as an example problem, consider the heliocentric time- and fuel-optimal transfers of a spacecraft from Earth to 3671 Dionysus, a small binary asteroid orbiting between the Earth and the asteroid belt. The optimal control problem for both time- and fuel-optimal trajectories was solved using an “in-house” code which utilizes arc-length continuation and homotopy techniques for improved and robust convergence. The formulation of the respective TPBVPs for both class of problems were used to obtain nominal transfer trajectories for a spacecraft with initial mass ($m_\text {0}$) of 4000 kg, equipped with an engine with a specific impulse ($I_\text {sp}$) of 3800 s and delivering a maximum thrust of $T_\text {max} =$ 0.33 N. Figures 1 and 2 depict the time-optimal and fuel-optimal nominal transfer trajectories respectively.

The crucial results characterizing the nominal trajectories are enlisted in Table 1 for both class of transfers. Note the trade-off between time of flight and $\Delta V$ requirements across continuous-thrust time-optimal transfer and the bang-off bang fuel-optimal transfer.

**Table 1:** Nominal trajectory parameters, time- and fuel-optimal.

Transfer type	Final mass ($m_f$), kg	Time of flight ($t_f$), yrs.	$\Delta V$ (km/s)
Time-optimal	2611.39	4.972	15.879
Fuel-optimal	2949.16	9.353	11.356

## Generation of extremal trajectory bundles

As discussed in the introduction, the primary objective of this work is to assess the capability of a supervised learning method based on Gaussian Process Regression to learn the structure underlying low-thrust transfer trajectories for both time- and fuel-optimal class of problems. This necessitates generation of a comprehensive database: ${\mathbb {D}} = \{{\mathbb {X}}(t), m(t),\varvec{\Gamma }(t), \lambda _m\}$, $t \in [t_0,t_f]$. The methodology used in the paper to generate ${\mathbb {D}}$ is based on the work of Izzo et al.^9^ on “backward generation of optimal examples”. A major stalling point in terms of database generation is that repeated solutions of hundreds or thousands of optimal control problems with underlying non-linear dynamics is a tedious task even with the most state-of the art continuation and homotopy tools accessible to mission designers as shown in some previous investigations^32–35^. Spacecraft motion re-planning as well as autonomous guidance using learning methods are applications that require predictions with a high-level of accuracy. Especially, for on-board re-planning of time-to go trajectories, the predictions provided for the unknown initial ad-joint n-tuple ($\varvec{\Lambda }(t_0)$) should preferably converge in a low-single digit number of iterations. The same database, ${\mathbb {D}}$, will be used for 1) Trajectory re-design/re-plan due to engine off-nominal thrust performance 2) Autonomous Guidance using best-estimate of the current spacecraft state (including navigation errors) and a desired target orbit. The following sections describe the methodology to populate ${\mathbb {D}}$ with a bundle of neighboring optimal trajectories terminating in the the desired orbit without solving repeated TPBVPs.

### Time-optimal trajectories

For a class of trajectories that are optimized to minimize time of flight, the engine is assumed to operate always on ‘full-throttle’. The control is therefore limited to effective thrust direction which according to Lawden’s Primer Vector theory^36^, lies in a direction opposite to the primer vector to minimize the Hamiltonian in order to achieve optimality. The indirect formulation of the optimal control problem leading to a TPBVP was discussed at length in “Trajectory optimization problem” section. The solution procedure typically requires a guess for the initial ad-joint n-tuple i.e., $\varvec{\Lambda }(t_0)$ and a shooting method is employed until the terminal orbit conditions as well as the associated transversality conditions (happens when any terminal state is left free). For the calculus of variations development of the indirect formalism leading to the first order necessary conditions for optimality and the transversality conditions, the reader is referred to^37^. The associated boundary conditions including the transversality conditions for time-optimal transfers arising due to the assumption of free $L_f$, free $t_f$ and free $m_f$ is,

22 $$\begin{aligned} \varvec{\psi } \ (\varvec{X}^{*}(t_f),t_f) = \bigg [[\varvec{X}^{*}(t_f) - \varvec{X}^{*}_\text {T}]^T, ||\varvec{\Lambda } (t_f)|| - 1, \lambda _L(t_f), \lambda _m(t_f)\bigg ]^T = \ \ {\mathbf {0}}, \end{aligned}$$

where $\varvec{X}^{*}_\text {T}$ is the vector of the target state conditions that have fixed desired values, $||\varvec{\Lambda } (t_f)|| - 1 = 0$ is an alternate transversality condition related to the free final time, which was used instead of the condition on $H_\text {T} (\text {t}_f)$^38^ and $\Big [\lambda _L(t_f), \lambda _m(t_f)\Big ] = [0,0]$ are transversality conditions associated with free final true longitude and free final mass respectively.

The target is to find a neighboring bundle of optimal trajectories around the nominal. These neighboring trajectories are required to satisfy the necessary conditions for optimality while also satisfying the transversality conditions. The converged values for the ad-joint n-tuple resulting in the nominal trajectory are perturbed in a small neighborhood along with a perturbation in the final mass of the spacecraft after flying the nominal trajectory. The perturbation cannot be arbitrary since, the transversality conditions must be satisfied in order to maintain consistency across the whole bundle. The algorithm for generating time-optimal bundle of extremals is outlined below,


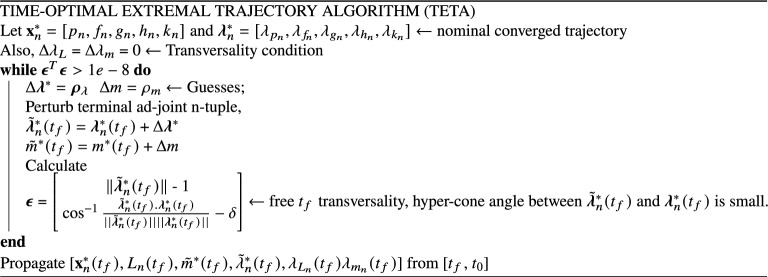


**Figure 3:**
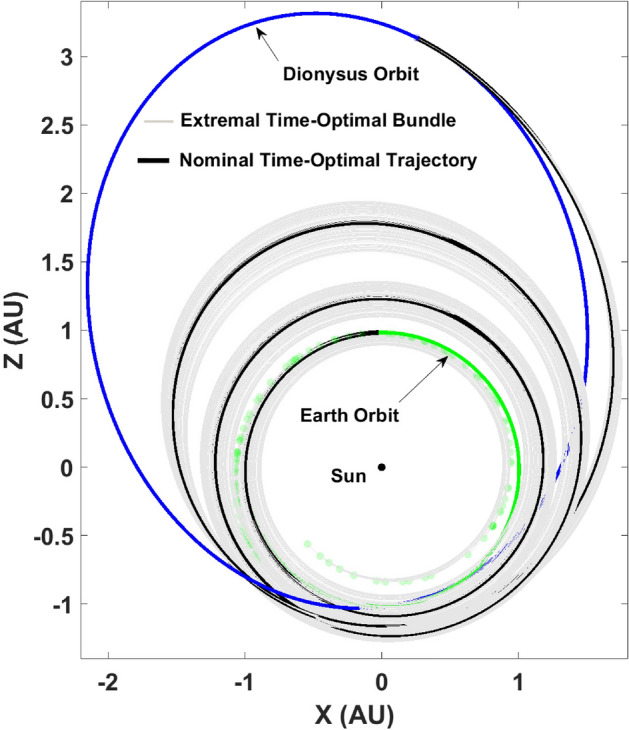
Time-optimal extremal bundle generated using TETA: $N = 100, \delta = 1 \times 10^{-3}$.

As the algorithm states, the Lagrange parameters related to the states: true longitude and mass are unperturbed since the nominal values satisfy the transversality conditions already. The remaining subset of the ad-joint n-tuple is perturbed along with the final mass and the resulting state-ad-joint set is propagated backwards in time from $t_f$ to $t_0$ in accordance with the respective ODEs describing the state-dynamics described previously as well as the ad-joint dynamics obtained from the Euler–Lagrange equations. Figure 3 depicts the extremal trajectory bundle consisting of 100 neighboring time-optimal trajectories.

### Fuel-optimal trajectories

**Figure 4:**
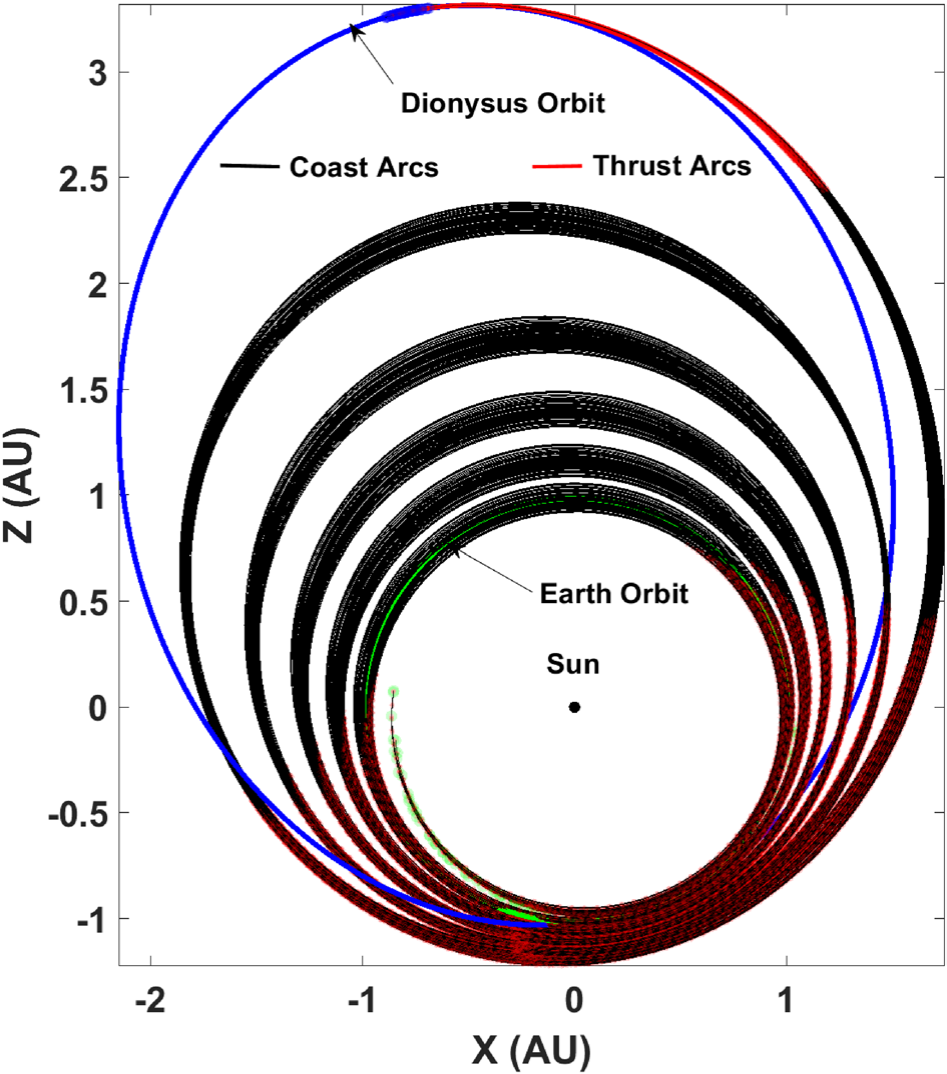
Fuel-optimal extremal bundle generated using FETA: $N = 100, \delta = 1 \times 10^{-3}$.


In the fuel-optimal case, the extremal bundle generation via the algorithm described above follows a slightly different path. Since, the fuel-optimal trajectories have thrust-coast-thrust segments embedded in the solution, the extremal bundle can be generated to have a free final true longitude. Therefore, $\Delta L (t_f)$ is obtained using a root finding numerical procedure by enforcing the transversality condition, $H_F(t_f) = 0$ given the nominal states and the perturbed costates. Note that the costates associated with true longitude and mass are still set as zero in accordance with the remaining transversality conditions associated with the respective free final states. Figure 4 depicts the fuel-optimal extremal bundle generated using FETA and consists of 100 neighboring fuel-optimal trajectories with $\delta = 1 \times 10^{-3}$.

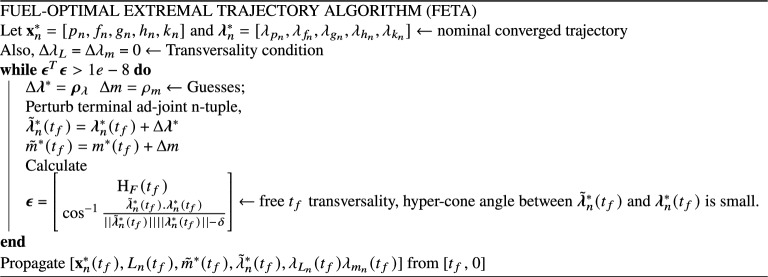


## Results: Trajectory re-planning—off-nominal thrust

A common source of off-nominal performance of a spacecraft stems from an off-nominal thruster performance. Essentially, the design of a nominal low-thrust trajectory is done based on the nominal ratings of the thruster performance depending on factors like maximum thrust and engine specific impulse. These parameters are bound to deviate from their nominal values during a mission. For instance, the NASA mission to exoplanets Ceres and Vesta had to be redesigned $> 100$ times.

Due to the requirement of redesigning and refining the nominal based on current engine performance parameters, solution methodology to obtain optimal trajectories must be robust. The obvious challenges associated with robust convergence to an optimal solution for multi-spiral, low-thrust trajectories is well-known and well documented in literature. This calls for a methodology which can provide rapid convergence based on the knowledge of the best known current spacecraft state and the desired final state as a solution to the underlying TPBVP. GPR provides a supervised learning tool which can be used to train a regression model based on the pre-computed neighboring extremal bundle to predict the unknown initial values of the ad-joint n-tuple accurately. This prediction can then be used to run the indirect optimal control algorithm and therefore, facilitate rapid numerical convergence.

**Figure 5:**
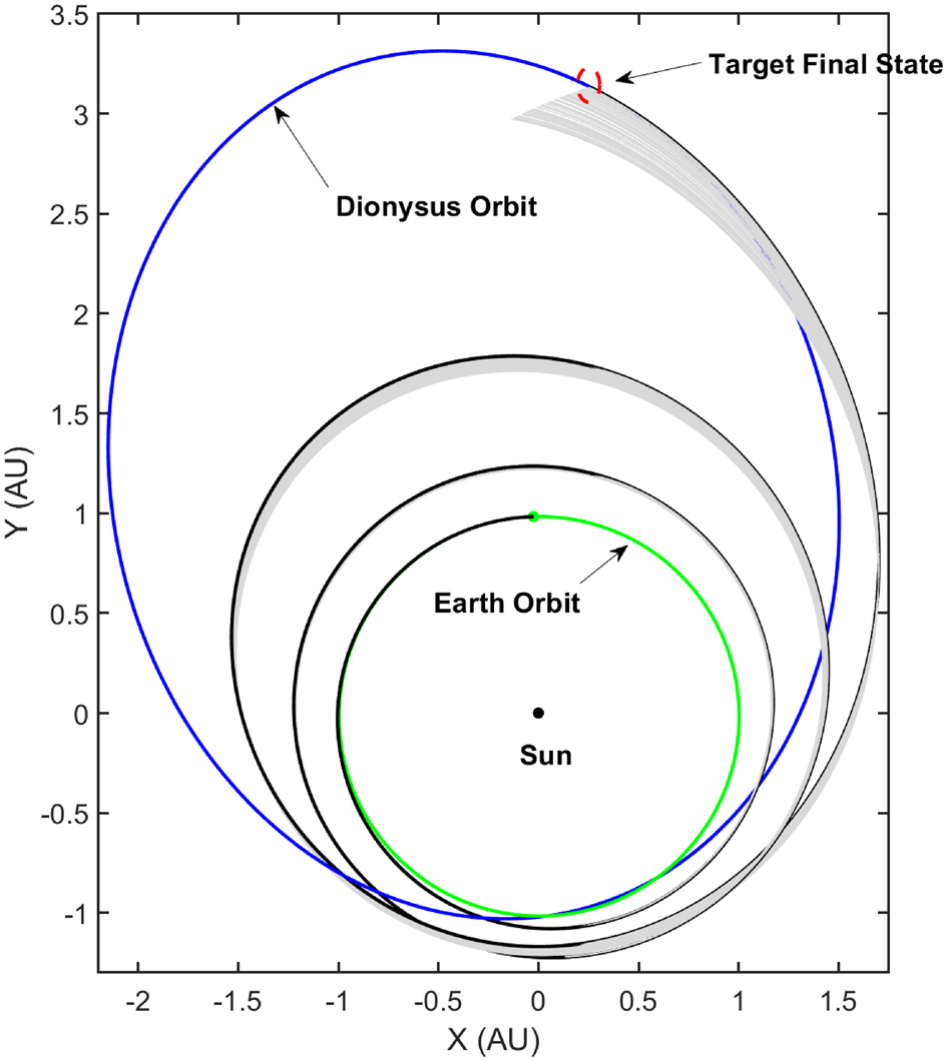
$5 \%$ knockdown bundle of non-optimal trajectories.

Consider the time-optimal trajectory of a spacecraft as depicted in Fig. 1 with nominal engine parameters listed in “Nominal trajectory” section. This is the nominal trajectory designed to achieve a minimum-time transfer from Earth to 3671 Dionysus. For the next step, assume an off-nominal performance which is described as a thrust knockdown within $5\%$ of the nominal, while still flying the nominal control profile i.e., the nominal primer vector profile defines the thrust steering angle profile. The propagation is defined as,

23 $$\begin{aligned} \text {Propagate}_{[{\mathbb {X}}_0, \varvec{\Lambda }_0^{*}, \text {T}_\text {max}^{\downarrow }]} [\dot{{\mathbb {X}}}^{T} \ \ \dot{\varvec{\Lambda }}^{T}\ \ {\dot{m}}^{T}]^{T} \ \ \text {from} \ \ [0,t_f] \end{aligned}$$

where $\text {T}_\text {max}^{\downarrow } = {\mathcal {U}}(0.95 \text {T}_\text {max}, \text {T}_\text {max})$ is sampled from a uniform distribution and denotes the knocked down thrust for the off-nominal engine. Note that other engine parameters are assumed constant. This results in a bundle of trajectories upon propagation with only one of the trajectories satisfying necessary optimality conditions, the nominal trajectory. Figure 5 depicts this bundle with the nominal trajectory highlighted in black. Note that the non-optimal trajectories, unsurprisingly, fail to satisfy the final boundary conditions due to the fact that the propagation uses the “optimal” initial ad-joint n-tuple which is no longer a solution to the updated TPBVP owing to the reduced thrust value.

For the sake of discussion, assume a scenario where the spacecraft has flown the non-optimal trajectory with an off-nominal thrust for $n_t = 500$ time steps before re-convergence is deemed necessary by the ground-based mission flight control team. A GPR model trained on the time-optimal extremal bundle can be used to predict the unknown initial ad-joint n-tuple with ‘initial’ signifying the $n_t$th time step. This prediction can then be used to run the indirect optimal control algorithm to re-solve the ‘to-go’ TPBVP for the remainder of the journey. If the prediction is robust to off-nominal thrust, such that convergence is achieved in a small single digit number of iterations, this methodology can serve as a game-changer for mission re-planning and operations.

There are a couple of important assumptions that need to be highlighted: (1) The ‘to-go’ TPBVP is still solved assuming nominal thrust. This means that the spacecraft will still diverge from the resulting converged trajectory. Thus, more frequent re-planning might be necessary depending on mission requirements and navigation capabilities. (2) Perfect navigation information is assumed to be available to the algorithm. The problem of uncertain spacecraft current state information will be tackled in later sections.

A MIMO GPR was trained using the data-points arising out of intersection of the hyperplane which signifies the $n_t$th time step. The states (MEEs and mass) were deemed as inputs whereas the ad-joint n-tuple was the output. The converged hyperparameters for the selected RQ kernel are listed in Table 2. Note that the initial guess for $log(\sqrt{\varvec{\Phi }})$ was $0.01 {\mathbb {I}}_{1\times 7}$, where ${\mathbb {I}}_{m \times n}$ denotes a matrix of ones of size $m \times n$.

**Table 2:** Converged hyperparameters: off-nominal thrust bundle GPR.

$log(l_p)$	$log(l_f)$	$log(l_g)$	$log(l_h)$	$log(l_k)$	$log(l_L)$	$log(l_m)$	$log(l_{\sigma _f})$	$log(\alpha )$
5.16595	0.01255	0.02018	0.01021	0.01097	0.39822	0.13573	-1.59750	1.31154

**Figure 6:**
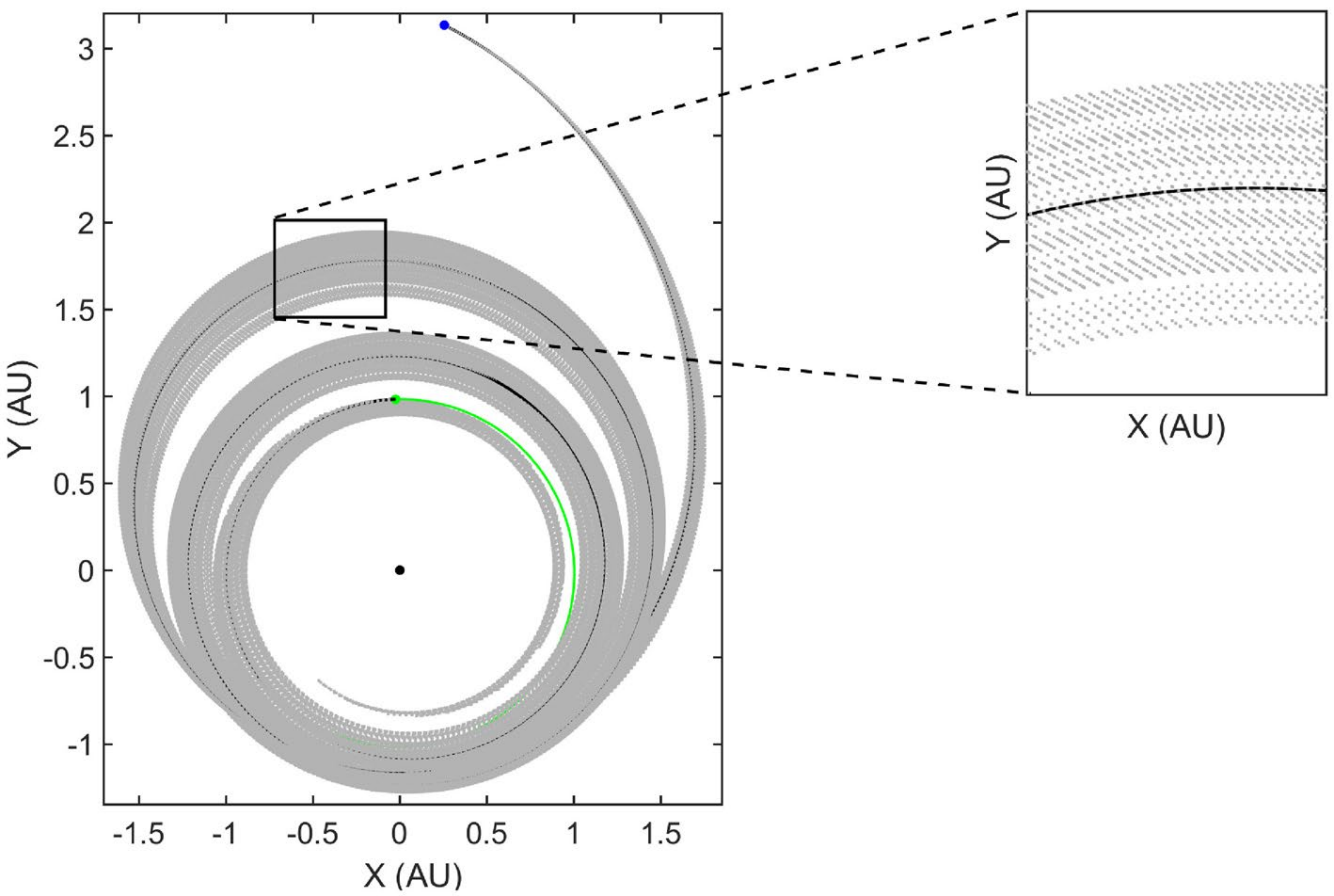
6D spacecraft states from the time-optimal bundle represented as time slices on a 3D plot.

The converged MIMO GPR was then used to re-plan the mission from the 500th time step to the end of the mission. Figure 6 depicts the time sliced states scattered around the nominal trajectory for all integration time steps of the time-optimal case.

**Figure 7:**
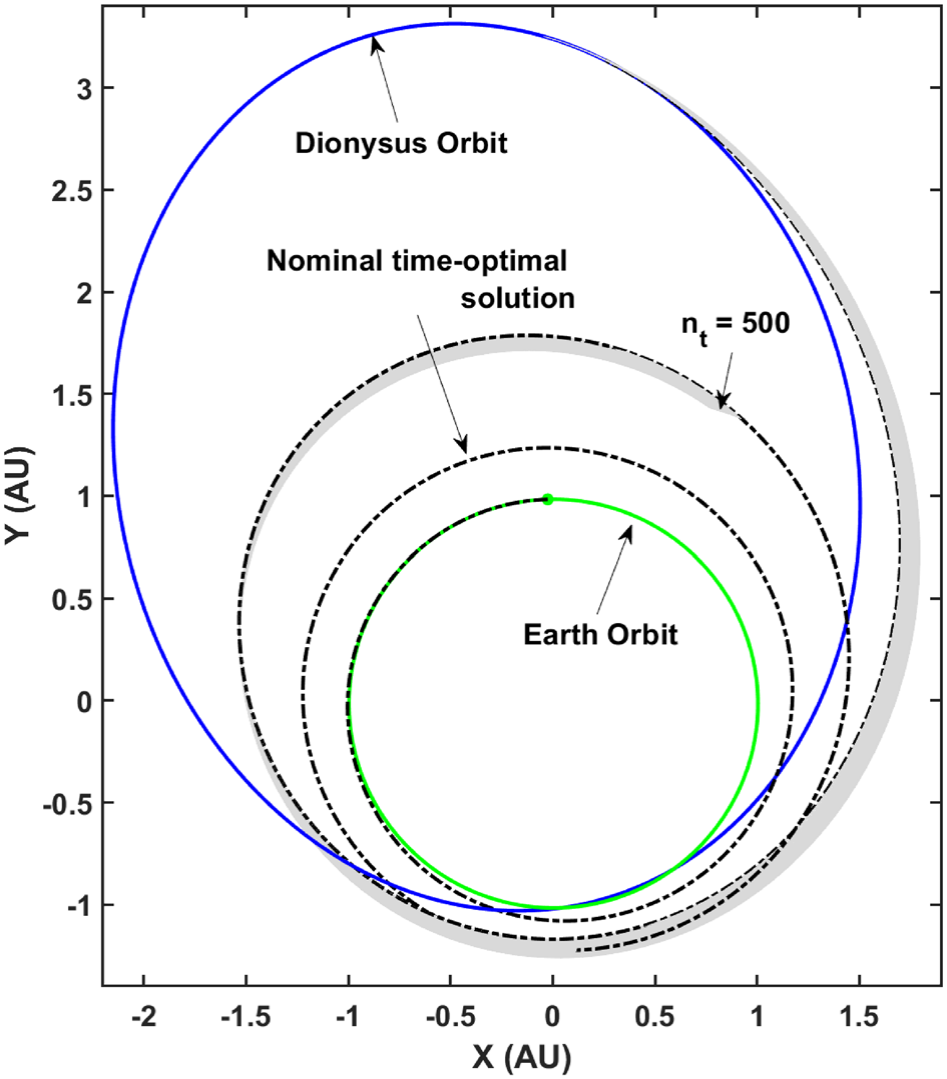
Re-converged bundle starting from the off-nominal states at $n_t = 500$.

Finally, the re-planned trajectory was computed after running the indirect optimal-control algorithm with the GPR prediction as the input for the unknown costate n-tuple. The trajectories were re-converged for all 100 cases in the off-nominal thrust bundle generated previously. The algorithm converged to the optimal ‘to-go’ solution in an average of 15 iterations with an average run-time of 0.03 seconds on a 64- bit Windows system equipped with Intel(R) Xeon(R) CPU @ 2.70 GHz and 16 GB memory. Figure 7 depicts the re-converged ‘to-go’ trajectories starting at the 500th integration time-step.

The robust and rapid convergence of the ‘to-go’ trajectories depict the strength of using a GPR based supervised learning methodology to train a regression model for robust and rapid re-planning of space missions for the time-optimal class of trajectories. Note that a similar procedure can be followed to re-plan fuel-optimal trajectories as well. An important consideration which was mentioned before was the divergence of the converged ‘to-go’ trajectories due to the off-nominal thruster performance in the future. This is due to the fact that the thruster performance is not a discrete phenomenon.

For real missions, the described regression based spacecraft flight control modelling would need to be performed at multiple time-steps during the entire mission. The frequency of this operation would depend on the following: (1) an acceptable maximum deviation from the nominal trajectory will govern the generated optimal bundle of trajectories (2) characterization of the off-nominal thruster performance and the subsequent future maximum deviations from the nominal. In essence, a more frequent re-planning operation based on the regression model is akin to autonomous guidance of the spacecraft.

**Figure 8:**
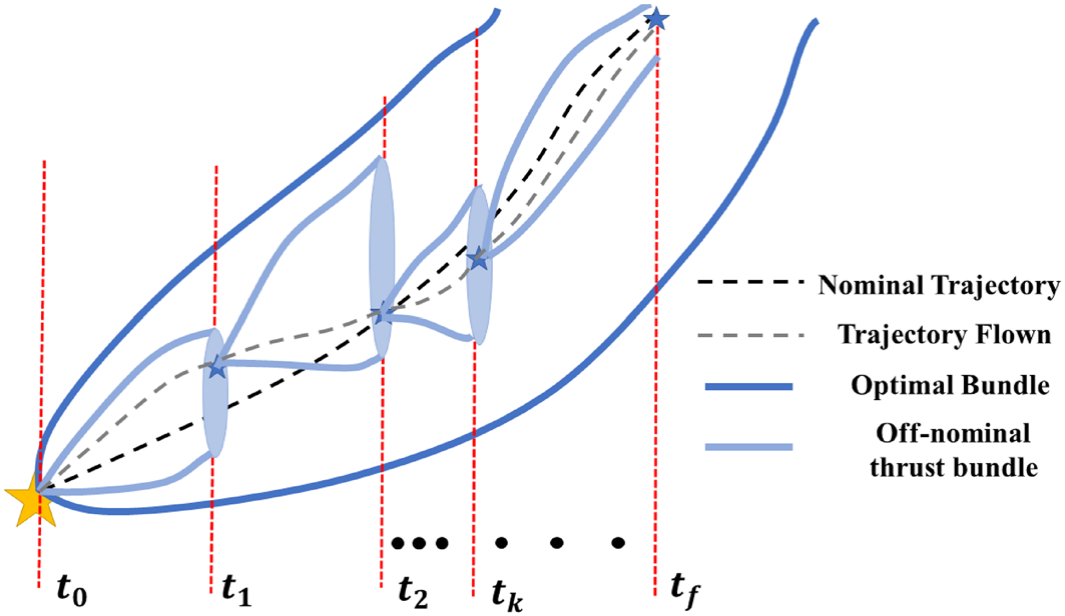
A schematic describing GPR based guidance of spacecraft with off-nominal thruster performance.

The schematic in Fig. 8 describes the concept of a GPR based autonomous guidance of a spacecraft with off-nominal thruster performance and with access to perfect navigation data succinctly. Assume that the time-optimal bundle, $\varvec{\zeta }_\text {op}$ has been generated based on some acceptable maximum 2-norm deviation from the nominal, $|| \delta _s||$. The spacecraft with states ${\mathbf {X}}_0$ at $t_0$ flies with an uncertain off-nominal thruster performance till $t_1$ with an associated smaller bundle. It is assumed that the 2-norm deviation in the off-nominal thrust bundle, $||\delta _\text {off}||$ is smaller than the selected value of $||\delta _s||$. At $t_1$, it is assumed that perfect navigation data is available and the trained MIMO GPR is used to predict the unknown ad-joint n-tuple and a subsequent optimal ‘to-go’ trajectory is generated. This trajectory is flown by the spacecraft to $t_2$ again with an off-nominal thruster performance, thereby diverging from the converged trajectory. A similar procedure is carried out at $t_2$ and then at subsequent time-steps until $t_f$ when the spacecraft reaches the desired final state ${\mathbf {X}}_f$.

An example implementation of the aforementioned GPR based autonomous guidance method is presented below. The off-nominal trajectory was assumed to have a fixed $3\%$ thrust knockdown from the nominal thruster performance and the re-planning occurred during the $N_t = [50, \ 250, \ 500, \ 600]^\text {th}$ time steps. Note that the initial/original propagation resulted in data with 1000 time-steps. The re-planning time-step has been limited to 600 because beyond this time-step, the off-nominal bundle begins to super-cede the time-optimal bundle generated via backward propagation and therefore the subsequently trained regression model performs poorly. Of course, this puts a limit on the receding horizon while implementing this methodology but since the actual trajectory is re-computed after every re-plan step the discussion above is only valid locally.

The procedure outlined before was followed with four respective regression models trained. It is worthwhile to highlight that since the regression model is trained on the optimal trajectory bundle, it requires generation of the data-set using the backward propagation algorithm only once.

**Figure 9:**
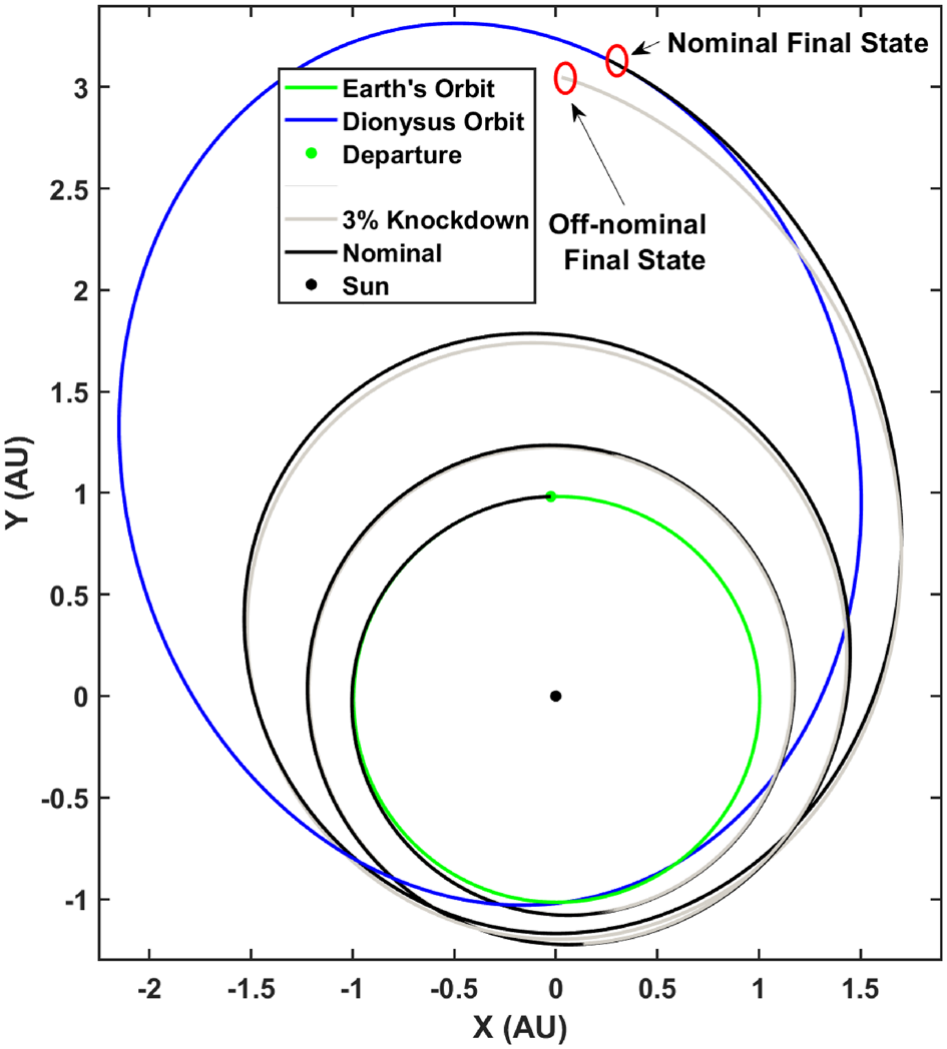
Nominal and $3\%$ knockdown trajectories.

Firstly, Fig. 9 depicts the nominal trajectory and the $3\%$ knockdown trajectory propagated with the nominal optimal control. The deviation in the final states is graphically demonstrated.

Next, four distinct MIMO GPRs were trained at the $N_t$ time-steps and the respective converged hyperparameters are listed in Table 3. Note that the initial guess for $log(\sqrt{\varvec{\Phi }})$ was the same as before i.e., $0.01 {\mathbb {I}}_{1\times 7}$.

**Table 3:** Converged hyperparameters: $N_t$ time steps.

$N_t$	$log(l_p)$	$log(l_f)$	$log(l_g)$	$log(l_h)$	$log(l_k)$	$log(l_L)$	$log(l_m)$	$log(l_{\sigma _f})$	$log(\alpha )$
50	5.49756	− 0.06079	− 0.05585	− 0.00570	− 0.01859	1.03146	− 0.00386	− 0.47098	0.73479
250	5.21997	− 0.03563	− 0.01211	0.00951	0.00661	1.43794	0.01848	− 1.69784	1.36503
500	5.16595	0.01255	0.02018	0.01021	0.01097	0.39822	0.13573	− 1.59750	1.31154
600	3.88893	0.01071	0.02242	0.01008	0.01059	0.02003	0.12542	− 0.89163	1.07042

**Figure 10:**
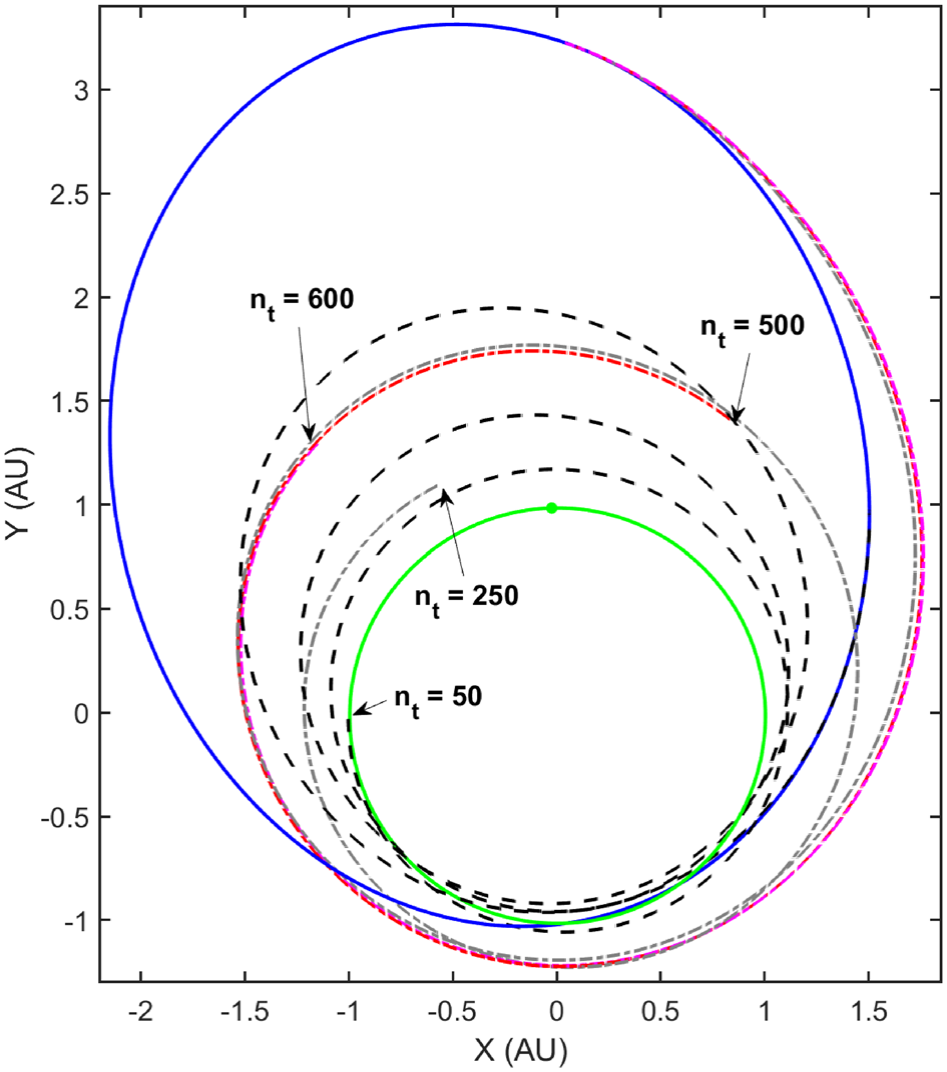
Independently Re-planned Trajectories starting at $N_t$ time-steps.

Figure 10 depict the re-converged ‘to-go’ trajectories with independent re-planning at the $N_t$ time-steps. It is hereby reiterated that these are the ‘to-go‘ trajectories where a re-plan has occurred at the $N_t$ time-steps independently. The thruster performance for these trajectories is assumed to be nominal.

**Figure 11:**
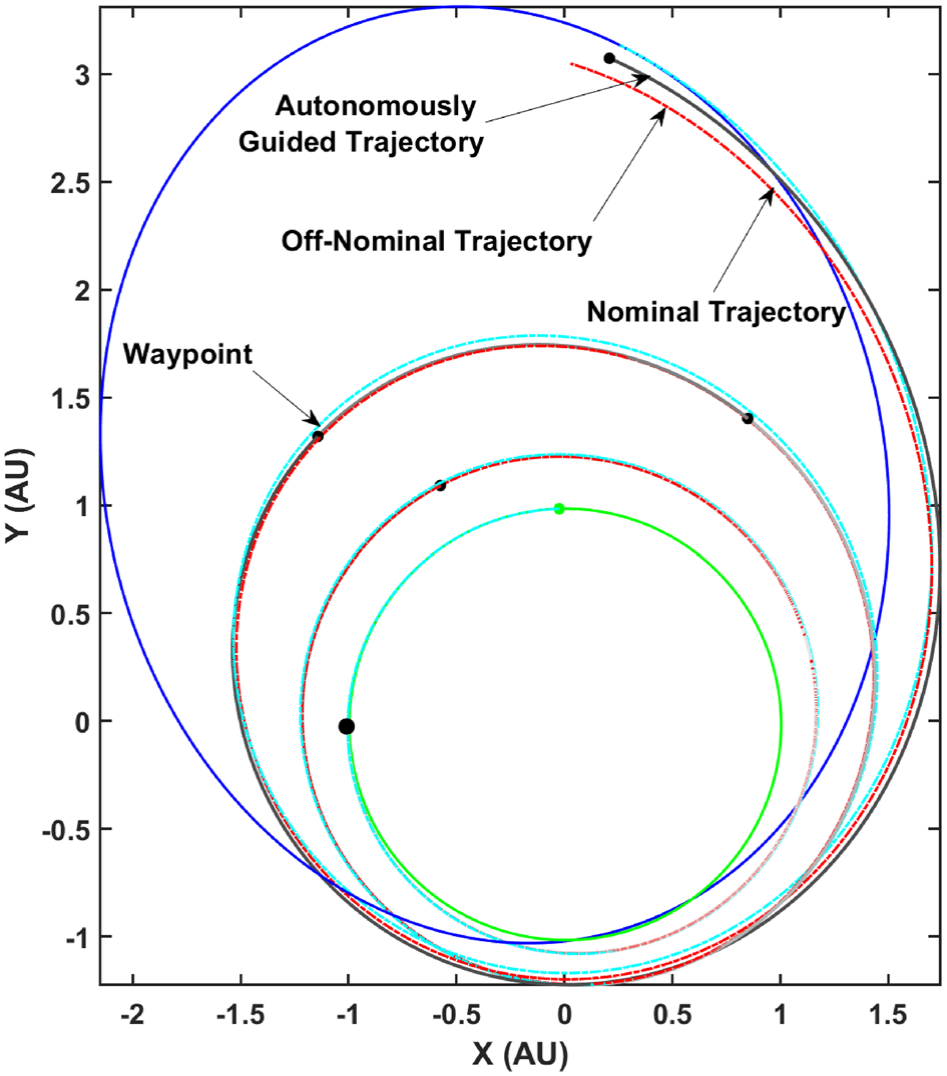
Autonomously guided trajectory with re-planning at the $N_t$ way-points.

Finally, the separate regression models are used to perform autonomous guidance of the spacecraft based on perfect navigation data available i.e., intermittent trajectories flown with off-nominal thruster performance is perfectly known. Figure 11 depicts the actual trajectory flown by the spacecraft by performing re-planning at the $N_t$ time-steps and therefore being autonomously guided towards the final target state. The black circles represent the intermediate way-points where a re-plan occurs and a new set of control parameters are computed by re-converging the trajectory. This trajectory is flown with off-nominal thrust to the next way-point and the process repeats.

It is immediately clear that the autonomously guided trajectory is graphically closer to the desired final state on the Dionysus heliocentric orbit by comparing it with the off-nominal and nominal trajectories. Note that this is only using four way-points i.e. the frequency of re-planning is four. A more accurately guided trajectory would require significantly more way-points to cancel out the errors accrued over time due to the off-nominal thrusting (a continuous disturbance). Heuristically, there are some hypotheses that may be considered, namely (1) firstly, it is hypothesized that more frequent corrections would lead to a more accurate guided trajectory (2) secondly, it is hypothesized that significantly frequent corrections would be required as the spacecraft approaches the target i.e., towards the end of the maneuver due to less time available for accomplishing the ‘to-go’ trajectory. Various case studies were performed to explore the validity of the aforementioned hypotheses. Table 4 lists the various parameters of the case studies.

**Table 4:** Case studies: autonomous guidance trajectories.

Case ID	Frequency	$N_t$
**A**	4	{50, 250, 500, 600}
**B**	6	{50, 250, 500, 700, 800, 900}
**C**	8	{50, 250, 500, 600, 700, 800, 900, 950}
**D**	8	{50, 500, 900, 950, 970, 985, 990, 995}

The cases were carefully crafted to effectively evaluate the claims of the hypotheses. A final 2-norm euclidean distance ($\Delta _{\mathbf {X}}, \Delta _{\mathbf {V}}$) between the guided spacecraft final position and velocity (${\mathbf {X}}_\text {guide}, {\mathbf {V}}_\text {guide}$) and the nominal trajectory final position and velocity (${\mathbf {X}}_\text {nom}, {\mathbf {V}}_\text {nom}$) was used as the metric to determine accuracy of the flown guided trajectory.

24 $$\begin{aligned} \Delta _{\mathbf {X}} = || {\mathbf {X}}_\text {nom} - {\mathbf {X}}_\text {guide}||_2, \end{aligned}$$

25 $$\begin{aligned} \Delta _{\mathbf {V}} = || {\mathbf {V}}_\text {nom} - {\mathbf {V}}_\text {guide}||_2 \end{aligned}$$

**Figure 12:**
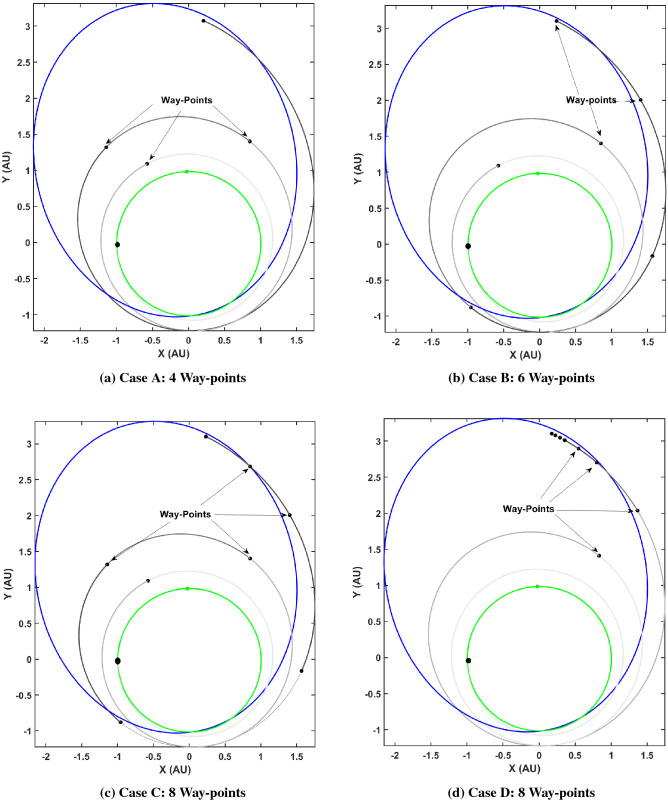
Case studies to explore the effect of frequency and location of way-points on guidance accuracy.

Figure 12 depicts the trajectory flown by the spacecraft for Cases A-D with re-convergence at respective way-points and Table 5 lists the $\Delta _{\mathbf {X}}$ and $\Delta _{\mathbf {V}}$ values for each case.

**Table 5:** Case studies: autonomous guidance trajectories.

Case ID	$\Delta _{\mathbf {X}}$ (DU)	$\Delta _{\mathbf {V}}$ (VU)
**A**	16.7163	$9.7 \times 10^{-3}$
**B**	8.7902	$1.3 \times 10^{-2}$
**C**	8.9869	$1.3 \times 10^{-2}$
**D**	18.6540	$1.4 \times 10^{-2}$

A comparison of cases A and B reveal that increasing the frequency of re-planning or in other words, increasing the number of way-points may result in a more accurate guided trajectory. On the other hand, a comparison of cases B and C show that this is not always true and the respective locations of the way-points also play a significant role. This is elucidated further by a comparison of cases A and D which show that with double the frequency but non-judiciously placed way-points, the accuracy deteriorates. Finally, a comparison of cases C and D reveal that for the same number of way-points, the relative positioning of the way-points can lead to starkly different performance of the autonomous guidance algorithm.

It must be mentioned here that the concept of incremental trajectory re-planning is analogous to Model Predictive Control (MPC) which has also been widely studied in the context of real-time space guidance applications. Eren et al.^39^ have covered the current state of research in this field with applications to aerospace systems quite comprehensively. The methodology presented in this work differs from the traditional MPC in that it uses the regression model trained on an extremal bundle of neighboring optimal trajectories to predict the unknown initial costates rather than starting with a random guess at each re-planning step. In the authors’ experience with solving TPBVPs originating from indirect approaches, the most challenging step is attributing an initial guess to the unknown and extremely ‘non-intuitive’ costates. The methodology presented and results discussed demonstrate that utilizing a regression model proves to be extremely effective in achieving rapid convergence even for multi-rev optimal trajectories.

## Conclusion

The issue of trajectory re-planning for long duration, multi-spiral, low-thrust trajectories is a long-standing problem in mission implementation. Historically, such long duration missions have been re-planned > 100s of times to account for unseen perturbations experienced by the spacecraft and therefore an unavoidable departure from the nominal. In this paper, a methodology equipped with associated algorithms to perform multiple trajectory re-planning of a new or an ongoing mission using on-board computers was presented. The methodology, based on a stochastic supervised learning approach, efficiently predicts the unknown costate n-tuple at the way-points which enables rapid and robust re-convergence of the ‘to-go’ trajectories. It was demonstrated that a cascade of way-points can then be used to form an autonomous guidance program and case studies were used to highlight the effect of frequency and location of way-points on the performance of the guidance algorithm for a spacecraft experiencing a fixed off-nominal thruster performance.

For future work, the methodology could be extended to include navigation errors. Secondly, the algorithm can be implemented for re-planning of fuel-optimal class of trajectories with slight modifications. Moreover, a useful addition would be inclusion of uncertainties in thruster performance and specific impulse of the spacecraft engine.

## Data Availability

The data-sets used and/or analysed during the current study available from the corresponding author on reasonable request.
